# The treatment of femoral neck fracture using VEGF-loaded nanographene coated internal fixation screws

**DOI:** 10.1371/journal.pone.0187447

**Published:** 2017-11-08

**Authors:** Shuo Li, Hengfeng Yuan, Jianfeng Pan, Wenshuai Fan, Liang Zhu, Zuoqin Yan, Changan Guo

**Affiliations:** Department of Orthopedics, Fudan University, Shanghai, China; University of Zaragoza, SPAIN

## Abstract

**Purpose:**

Previous studies have proved that vascular endothelial growth factor (VEGF) has a dual role in the promotion of new bone formation and blood vessel repair during fracture healing. However, how to introduce VEGF to a fracture site safely and effectively is still a challenge. This study aimed to prepare a VEGF-loaded nanographene coated internal fixation screw and to evaluate its effects in the treatment of femoral neck fracture.

**Methods:**

Nanographene coated screws were prepared by direct liquid-phase exfoliation of the graphite method, and the surface characteristics were observed through scanning electron microscopy (SEM). VEGF was loaded on nanographene coatings through physical adsorption, and the VEGF controlled release was examined by ELISA. Then a canine femoral neck fracture model was built to examine both the angiogenic and osteogenic properties of the VEGF-loaded coated screws. X-ray, micro-CT-based microangiography, and histopathologic evaluation were used to assess the fracture healing progress.

**Results:**

The results demonstrated that nanographene could load VEGF effectively, and the accumulative release of VEGF clearly increased during the entire testing period (9 days) without burst release. In canine fracture models, the results of X-ray, microangiography, and histopathologic examination proved that the speed of fracture healing, new bone formation area, and revascularization of the fractured femoral heads in the VEGF-loaded coated screws groups were significantly higher than in the control groups.

**Conclusion:**

Our study proved that VEGF-loaded nanographene coated screws were effective in the treatment of femoral neck fracture and prevention of avascular necrosis of femoral head.

## Introduction

Femoral neck fracture has two major complications, avascular necrosis of femoral head and the non-union of the fracture. While the rate of non-union recently decreased apparently with the improvement of the internal fixation techniques, the rate of ANFH remains the same [1]. The occurrence of ANFH is primarily related to its unique anatomical structure, the artery circle of branches of the medial circumflex femoral artery and lateral femoral circumflex artery [2]. ANFH may occur if the femoral head blood supply is not restored in a timely manner after fracture [3]. The application of vascular repair-related growth factors is now considered to be a more effective way to treat femoral neck fracture and reduce the incidence of ANFH because of its ability to promote early recovery of the blood supply of femoral heads after fracture [4,5].

Vascular endothelial growth factor (VEGF) is currently considered the most important regulatory factor of angiogenesis involved in various vascular repair processes [6,7]. It plays an essential role in fracture healing [5,8–11]. In the inflammatory phase of fracture healing, VEGF can recruit macrophages to the fracture site and regulate angiogenesis. VEGF participates in the regulation of the blood vessels and bone formation in intramembranous ossification and endochondral bone ingrowth in endochondral ossification. Given the important role of VEGF in both bone repair and vascular repair, we plan to load VEGF safely and effectively into the femoral head and hope it can be effective in the treatment of femoral neck fracture and the prevention of ANFH.

As the thinnest nanomaterial, graphene has only a single atomic layer of thickness and can be applied as a coating on any material surface [12]. Compared with other nanomaterials, graphene has an extremely high specific surface area, and both sides can absorb drugs and achieve high-dose drug loading [13–15]. In our study, we applied VEGF-loaded nanographene coated screws to the treatment of femoral neck fracture and evaluated whether it can simultaneously promote osteogenesis and angiogenesis in fracture healing.

In the evaluation of experimental results, we used a new contrast agent for the first time—subnanometer barium sulfate for canine femoral microangiography. After the image reconstruction of micro-CT, the evaluation of bony and vascular repair in fracture healing can be analyzed from two-dimensional and three-dimensional levels.

## Materials and methods

### Preparation of nanographene coated internal fixation screws

Nanographene was prepared by the direct liquid-phase exfoliation of graphite. Fifteen milligrams of natural graphite was mixed with 5 ml disperse solvent (1:1 isopropyl alcohol/H_2_O) in a 7.5-ml glass flask. After agitation for 10 minutes, the suspension underwent ultrasonic treatment for 4 hours (ultrasonic frequency, 40 kHz). The dispersion was centrifuged twice for 10 minutes at 1000 rpm at room temperature. The supernatant was collected and a stable graphene suspension was finally obtained. The internal fixation screws (Ti6Al4V, Wego, China) were immersed in nitric acid solution for 1 week in advance and then soaked in graphene supernatant for another week. After that, screws were removed and adequately rinsed by deionized water. The nanographene coated screws were prepared completely. The morphology of the surface of the coated screws was examined by scanning electron microscopy (SEM, JEOL Japan).

### In vitro cytotoxicity evaluation

The extracts of the nanographene coated screws were prepared according to ISO 10993–12. Steps are briefly described as follows. Sterile nanographene coated fixation screws were completely immersed in 6 ml endothelial cell medium (ECM) with 5% fetal bovine serum (FBS, Gibco, Thermo Fisher Scientific, Waltham, MA, USA) and osteoblast medium (ScienCell, San Diego, CA, USA) and placed in an incubator at 37°C for 72 hours. Coated screws were removed from the ECM and osteoblast medium, and the extracts were centrifuged for 5 minutes at 1000 rpm. The supernatants were sterilized through a 0.22-μm filter and refrigerated at 4°C and used within 1 week. The human umbilical vein endothelial cells (HUVECs) (Allcells, Alameda, CA, USA) and osteoblasts (Type culture collection of the Chinese Academy of Sciences, Shanghai, China) were used to evaluate the cytotoxicity of the coated screws. HUVECs and osteoblasts were seeded at a density of 3 × 10^3^/well in 96-well plates and cultured with 100 μl coated screw extract or special medium as control groups, for 4 days. The cells were then stained every 24 hours with 10 μl Alamar Blue dye and examined by microplate reader (wavelength, 450 nm).Each group was measured with n = 6 samples for the in vitro cytotoxicity evaluation, with 3 repeat measurements.

### VEGF loading and in vitro release kinetics

The 1-cm thread side of the nanographene coated screws and screws without coating were immersed in 20 ug/ml VEGF solution (dissolved in phosphate-buffered saline [PBS]) for 12 hours at 4°C. As the control group, the same length nanographene coated screws were immersed in phosphate-buffered saline (PBS) for 12 hours at 4°C.The screws were then washed three times with distilled water, lyophilized, and placed in a 15-ml sterile tube. The tube was added with 10 ml PBS (pH 7.4) and placed in a shaking water bath at 37°C and shaken at 100 rpm/min. At predetermined time periods (0 h, 8 h, 12 h, 24 h, 48 h, 72 h, 168 h, 216 h, and 288 h), 1 ml of supernatant was collected and replaced with the same volume of fresh PBS. The amount of VEGF released into the supernatant at each time point was measured by ELISA. Each group was measured with n = 3 samples for the in vitro release kinetics, with 3 repeat measurements.

### Released VEGF biological activity test

A VEGF-loaded nanographene coated screw was dissolved in 10 ml ECM (ScienCell, Carlsbad, CA, USA) with 5% FBS (Gibco) and placed in the same VEGF releasing condition in step 2.3 for 9 days. The supernatant was collected, sterilized by a 0.22-μm filter, and stored at 4°C (used within 1 week); 11.6% Matrigel (Becton, Dickinson, and Company, UK) was thawed at 4°C overnight. Ninety-six-well plates and micropipette tips were precooled on ice. In each well, 50 μl Matrigel was added and incubated at 37°C for 30 minutes to solidify; 4,000/100 μl HUVECs were added on the Matrigel and incubated at 37°C in an atmosphere of 5% CO_2_ and 95% air. After 12 hours of incubation, the HUVECs were imaged using an inverted microscope (Leica, Wetzlar, Germany). Three fields were chosen at random, and the length of the capillary-like tubes was measured by Image-Pro Plus 6.0 software.

### Animal experiment design and surgical procedure

All experimental animal surgery procedures and feeding standards were approved in accordance with the guidelines of the Care and Use of Laboratory Animals of the Zhongshan Hospital of Fudan University, Shanghai, China. Ten healthy adult (male and female) Beagle dogs, weighing 15–18 kg, were provided by the Laboratory Animal Center of Zhongshan Hospital of Fudan University. They were housed individually in the same room and could move freely in cages under conditions at 18–25°C,25–75% humidity, and 12h lighting (6:00–18:00). The room and cages were cleaned daily. And during the cage cleaning time, the dogs were taken outside of cages the reduce their stress. Animals were provied with 230-250g of solid food daily and filtered tap water and libitum. The operations would only be performed while the animal had no fear appearance, ate regularly and slept well. The left and right posterior limbs were treated with VEGF-loaded nanographene coated screws (experimental side) and nanographene coated screws without VEGF (control side) separately on the same dog. All surgery procedures were performed under basic anesthesia by injecting 10% pentobarbital sodium (25 mg/kg) intravenously. The fracture procedures are briefly described as follows. After general sterilization, a curved incision, approximately 10 cm, was made in the hip followed by the subcutaneous soft tissue separated step by step. The hip joint capsule was exposed and opened. Then a chisel was used to create a fracture at the midpoint of the femoral neck. The fracture was reduced and fixed temporarily with a 1.5-mm Kirschner wire. Then a VEGF-loaded nanographene coated screw was implanted into the femoral head through femoral torque and the other femoral head with a nanographene coated screw that was not VEGF loaded. All operative procedures were performed by the same surgeons. Postoperatively, all experimental dogs received routine care and were injected penicillin (800,000 units) intramuscularly for 3 successive days to prevent infection. For pain relief, meloxicam (0.2 mg/kg on the day of surgery, and there after the dose of 0.1 mg / kg every day for 1 week). General well-being, wound healing, and postoperative complications were observed daily. Sutures were removed after 2 weeks.

### Radiological examination

Under basic anesthesia, hip anteroposterior X-ray examinations were performed on all experiment animals at day 1 and at 8 and 12 weeks post surgery. The radiographic results were assessed by two radiologists who were blinded to the study.

### Microangiography

Preparation of the contrast agent consisted of 4% gelatin–30% subnanometer barium sulfate suspension. 1) 4% gelatin solution:120 g gelatin (Sigma) was added in 3000 ml PBS and placed in a shaking water bath at 37°C for 12 hours; 2) 900 g subnanometer barium sulfate was added in 3000 ml 4% gelatin solution and placed in a shaking water bath at 45°C for 1 hour; and 3) the suspension in step 2 was filtered three times by sterile gauze and used within 24 hours.

Under basic anesthesia, the dogs were fixed on the operating table in the dorsal position after skin preparation. A median abdominal incision (approximately 15 cm) was made followed by the skin, linea alba, and retroperitoneal tissue cut in a step-by-step fashion. The abdominal aorta and inferior vena cava were exposed and inserted with a vessel cannula, respectively. Two hundred and fifty milliliters of 0.9% saline with 12,500 U heparin was injected into the abdominal aorta for the heparinization of lower limb blood supply. Then, approximately 3000–5000 ml saline was injected into the abdominal aorta to replace the lower limb blood. After that, the experiment animals were sacrificed by injecting an overdose of anesthetics. One thousand milliliters of 4% paraformaldehyde was inputted into the abdominal aorta slowly. The 4% gelatin–30% subnanometer barium sulfate suspension was injected into the abdominal aorta rapidly with certain pressure. The infusion was stopped when there was an outflow of white liquid on the vena cava side. Then the abdominal aorta and vena cava were closed. To solidify the contrast agent sufficiently, all of the specimens were stored at 4°C overnight. The femoral heads were removed and fixed in 4% paraformaldehyde for 1 week at 4°C before micro-CT scanning.

### Micro-CT scanning and image reconstruction

After calibration, micro-CT (Healthcare Locus SP, General Electric, Fairfield, Connecticut, USA) scanning (46 μm resolution, scanning time 18 minutes) was performed on specimens. Reconstructed images, maximum-intensity projection (MIP), and Isosurface images were generated by Micro View software (General Electric). MIP is an overall projection of the femoral head that can display the whole two-dimensional distribution of vessels within the femoral head, whereas Isosurface exhibits a three-dimensional image. By selecting the appropriate threshold (3000 HU), the data of vessels within the femoral head can be extracted and reconstructed to a three-dimensional angiographic image through Isosurface.

After micro-CT scanning, the specimens were cut in half and then embedded in paraffin for histopathological analysis.

### Analysis of microstructure and bone mineral density of femoral head

**Fig 1:**
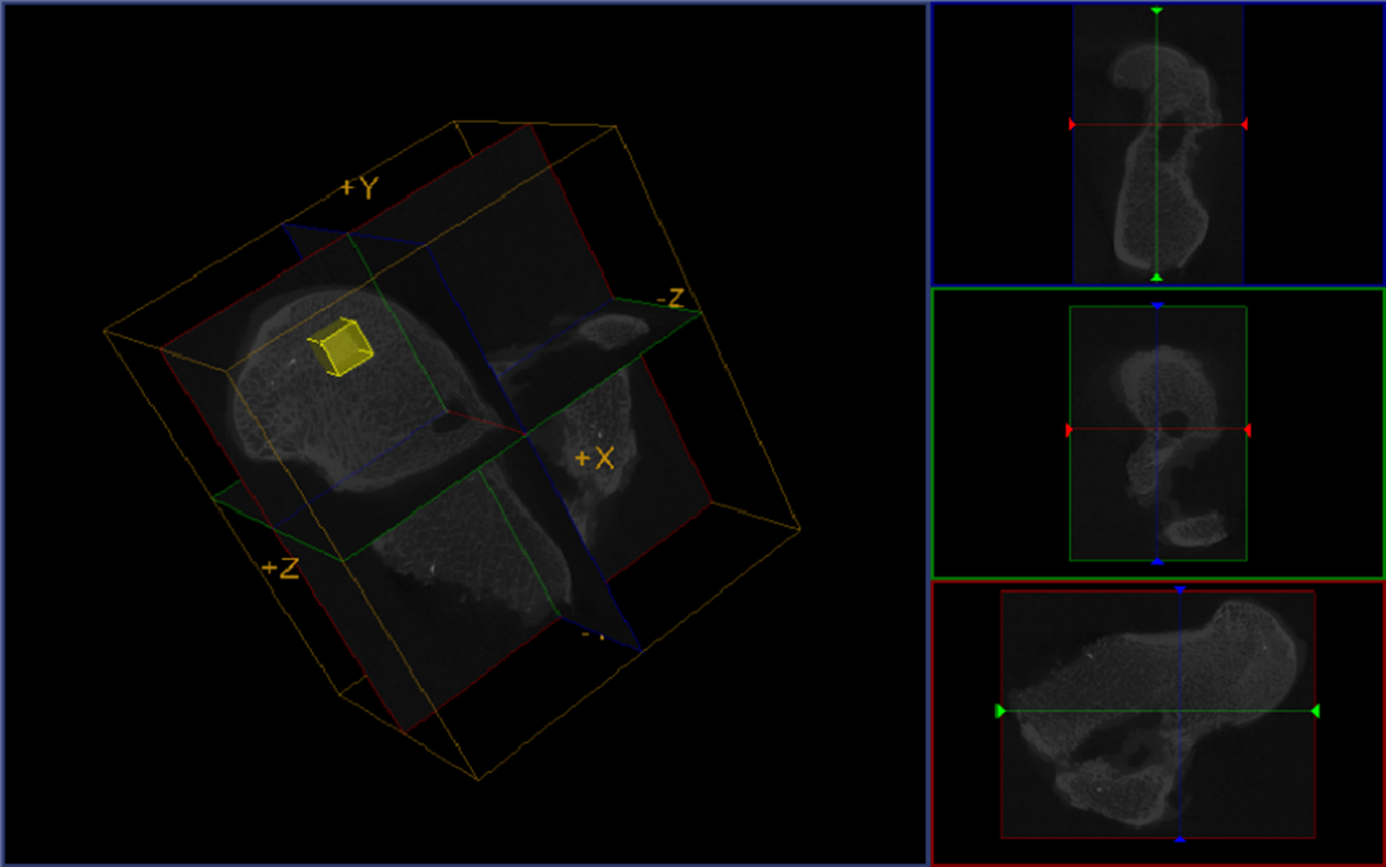
Advaned bone analysis (ABA) by Micro View software. After micro-CT image reconstruction, Micro View could analyze the BMD and microstructure of any certain volume of ROI within the femoral heads.

By manually choosing the uniform region of interest (ROI) (**Fig 1**), the trabecular thickness (Tb.Th) and bone mineral density (BMD) of femoral head, excluding the screw canal and cortical bone, were quantitatively analyzed by advanced bone analysis (ABA) in Micro View software. Each group was measured with 5 ROI for further analysis, with 3 repeat measurements.

### Histopathological evaluation

Dogs were sacrificed at 12 weeks after microangiography examination by injecting an overdose of pentobarbital sodium intravenously. Samples of bilateral femoral heads were harvested and fixed in 4% neutral-buffered formalin, decalcified in 10% ethylenediaminetetraacetic acid (EDTA) for further histopathological measurements after the surrounding soft tissue was removed clearly. Then the samples were sectioned and stained using hematoxylin and eosin staining (HE), Masson’s trichrome, and CD31 immunohistochemical staining. Stained sections were examined under an inverted microscope. Average Tb.Th, newly formed bone area (%), and microvessel density (MVD) [16] were obtained using Image-Pro Plus 6.0 and analyzed by two blinded pathologists.

### Statistics

All data are expressed as mean ± standard deviation (x±s). Statistical analysis was performed using SPSS 16.0 statistical software. Differences between the experimental and control groups were compared using an independent samples t test. P<0.05 was defined as statistically significant.

## Results

### Nanographene coated screws morphological observation

**Fig 2:**
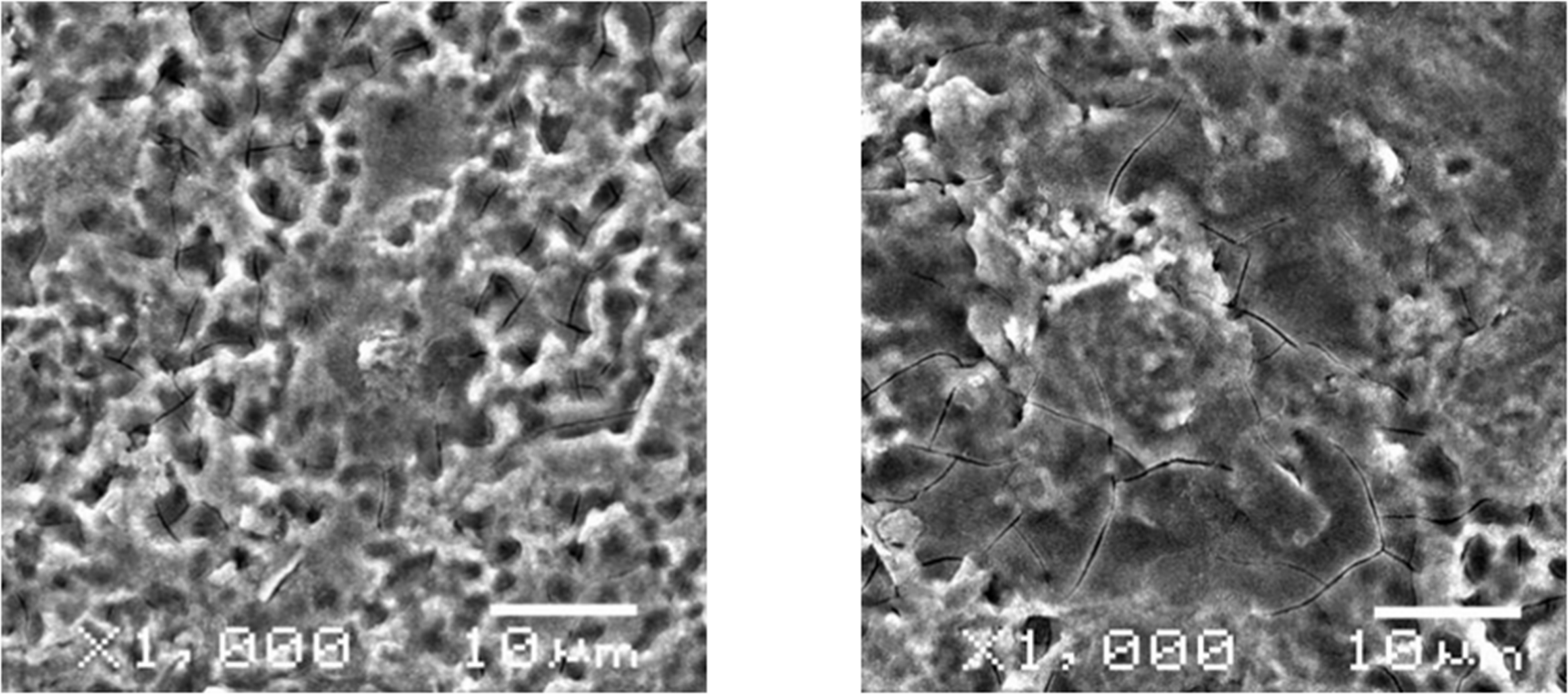
SEM images of internal fixation screws. The surface of nanographene coated screws (right) is covered with a thin layer of laminated material, and the morphology of the surface had clearly not been changed, as shown in the scanning electron microscopy (x1000).

SEM images of internal fixation screws with and without nanographene coating were obtained at 1000x magnification (**Fig 2**). The surface of screws without coating was performed as a loose and porous structure. After coating of the screws with nanographene, a thin layer of laminated material could be observed on the surface of the screws. Nanographene coating did not have an obvious change on the morphology of the screws.

### In vitro cytotoxicity evaluation

**Fig 3:**
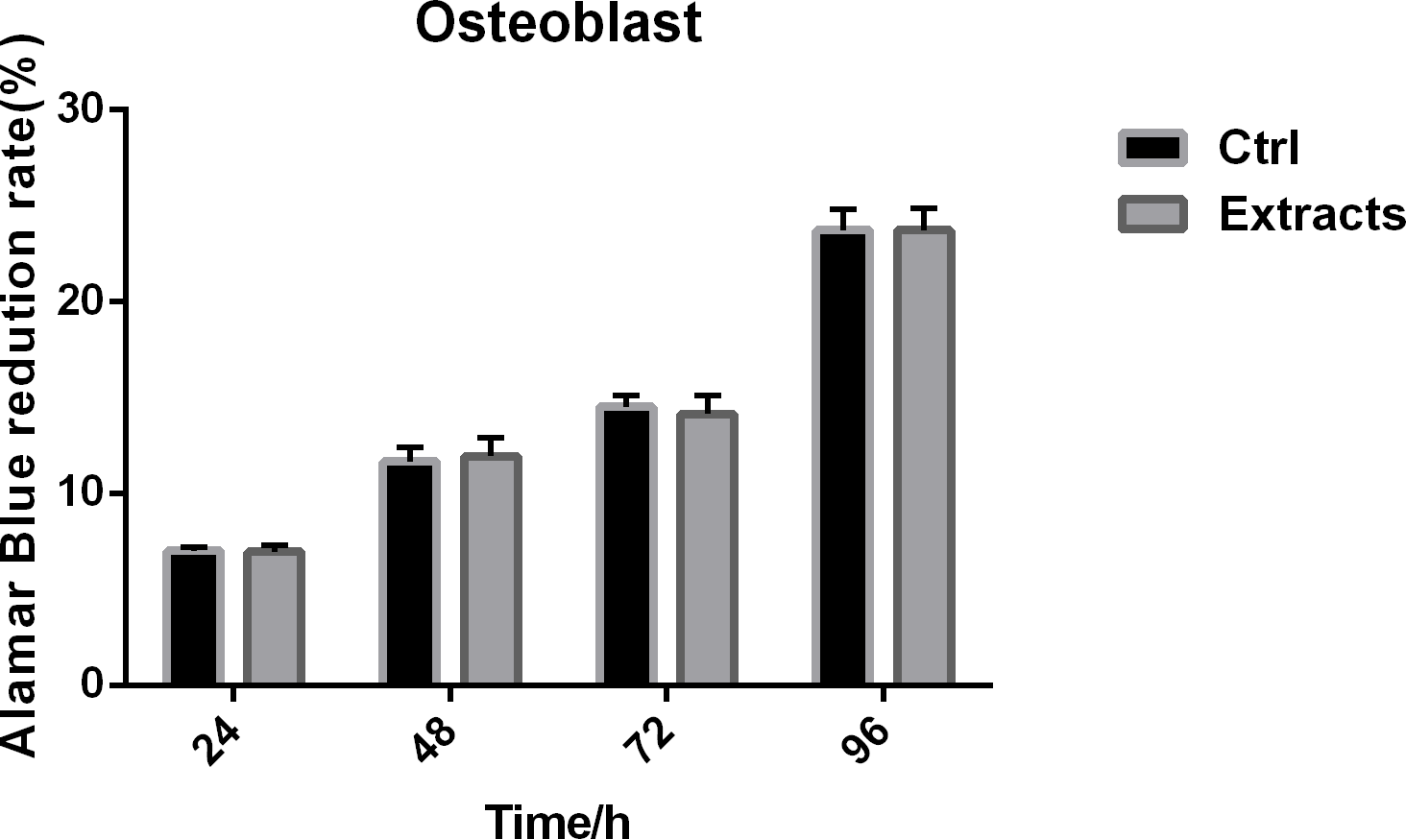
Osteoblast cytotoxicity test of nanographene coated screws. The result of the cytotoxicity test of nanographene coated screws showed that nanographene coating did not affect the biocompatibility of screws, because there was no significant difference between the control group and experimental groups.

**Fig 4:**
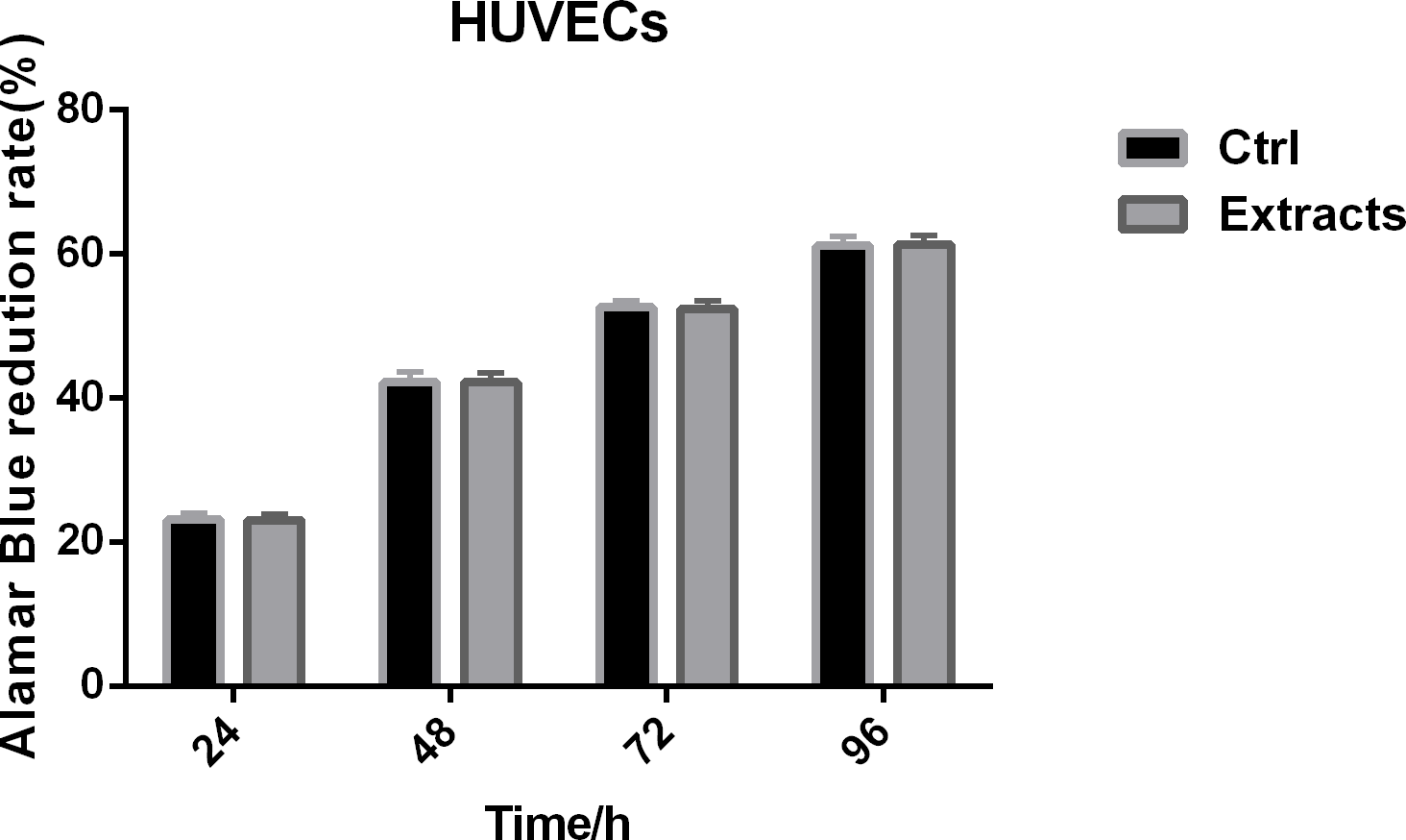
HUVECs cytotoxicity test of nanographene coated screws. The result of HUVECs cytotoxicity test showed that there was no significant difference between the control group and experimental groups as well.

A comparison of the cytotoxicity of the coated screw extracts with ECM or osteoblast medium were carried out by Alamar blue assay. As shown in **Figs 3 and 4**, there was no significant difference between the proliferation rate of the study groups (P>0.05). The results indicated that nanographene coating did not increase the cytotoxicity of the internal fixation screws.

### In vitro release kinetics

**Fig 5:**
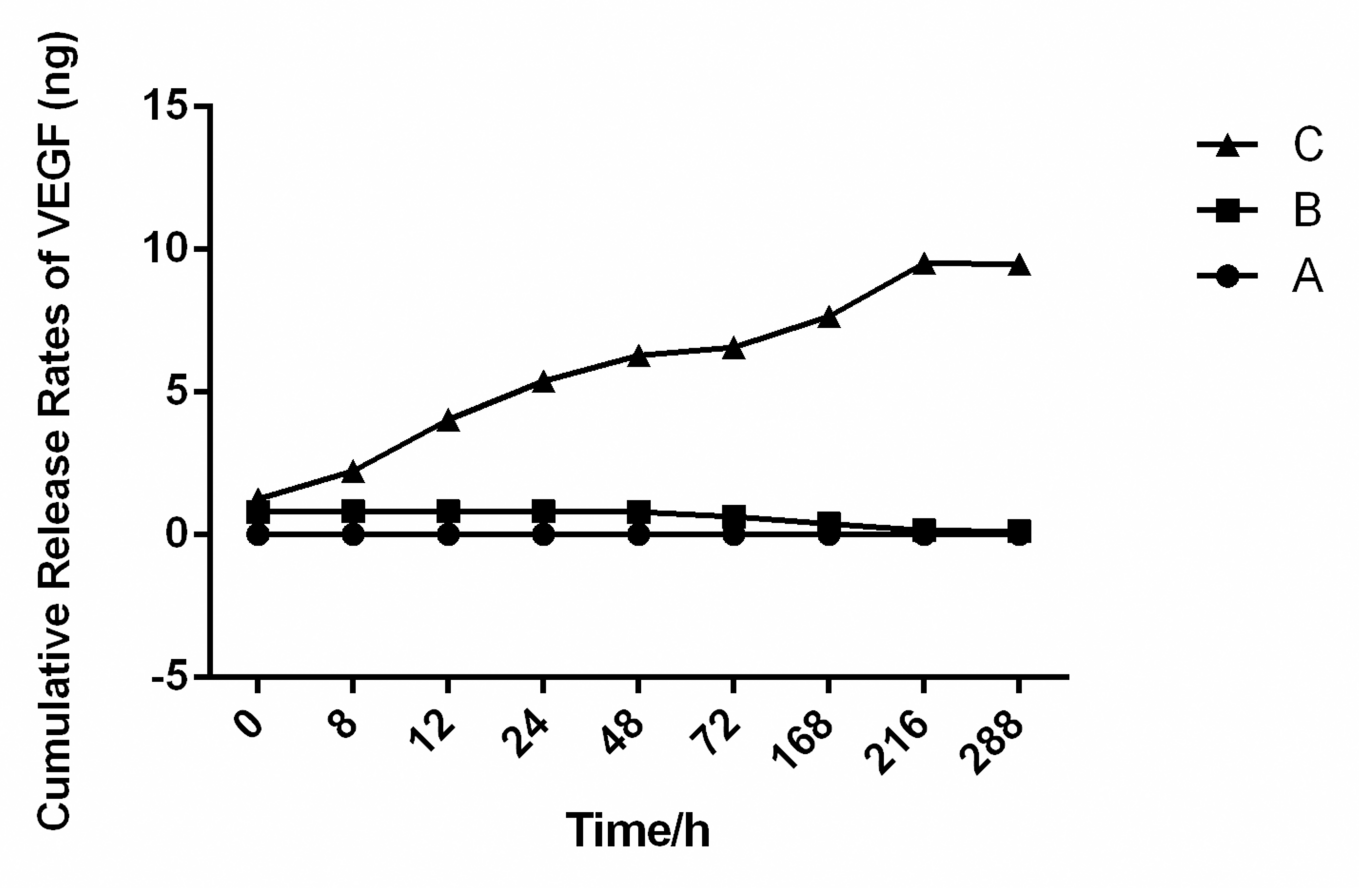
Release curve of VEGF. The curve (C) showed that the sustained release of VEGF on nanographene coating could last 9 days without a burst release. As the control gourp (B), nanographene coating screws without VEGF loaded had no VEGF released. There had very little VEGF burst relese on screws without nanographene coating (A) had no sustainded release effect.

The cumulative release rates of VEGF from nanographene coated screws in PBS are shown in **Fig 5** and **Table 1**. The release of VEGF was sustained for 9 days with a cumulative release amount up to 9.51 ± 0.23 ng. There was no burst release observed in the whole process. In the control group, there had very little burst release of VEGF in the uncoated screws in the first few hours and degraded within 3 days.

**Table 1:** The amount of sustained-release VEGF in three different groups(n = 3). ($\bar{x}$±s).

**Time(h)**	**0**	**8**	**12**	**24**	**48**
**Uncoated screws (ng)**	0.01±0.00	0.01±0.01	0.01±0.01	0.00±0.00	0.01±0.01
**Coated screws with VEGF (ng)**	0.79±0.07	0.81±0.12	0.80±0.11	0.80±0.13	0.79±0.10
**Coated screws w/o VEGF (ng)**	1.09±0.19	2.02±0.28	4.07±0.27	5.20±0.27	6.21±0.20
**Time(h)**	**72**	**168**	**216**	**288**	
**Uncoated screws (ng)**	0.00±0.00	0.01±0.01	0.00±0.00	0.00±0.00	
**Coated screws with VEGF (ng)**	0.62±0.06	0.37±0.04	0.15±0.02	0.10±0.01	
**Coated screws w/o VEGF (ng)**	6.68±0.38	7.76±0.40	9.51±0.23	9.47±0.13	

The release of VEGF was sustained for 9 days with a cumulative release amount up to 9.51 ± 0.23 ng. There was no burst release observed in the whole process. In the control group, there had very little burst release of VEGF in the uncoated screws in the first few hours and degraded within 3 days.

### Released VEGF promotes angiogenesis in vitro

**Fig 6:**
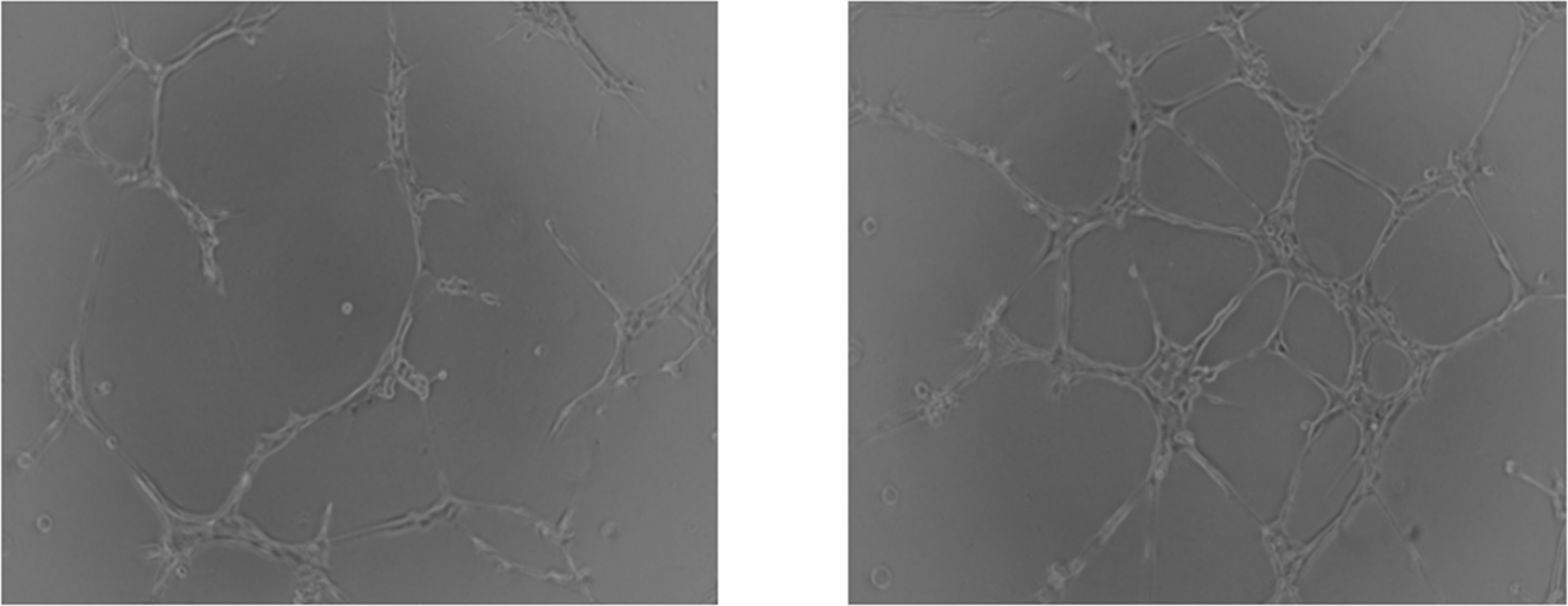
The biological activity of released VEGF. The cumulative sustained release of VEGF within 9 days still had biological activity and could promote the tube formation of HUVECs (right) compared with the control groups (left) in vitro.

**Fig 7:**
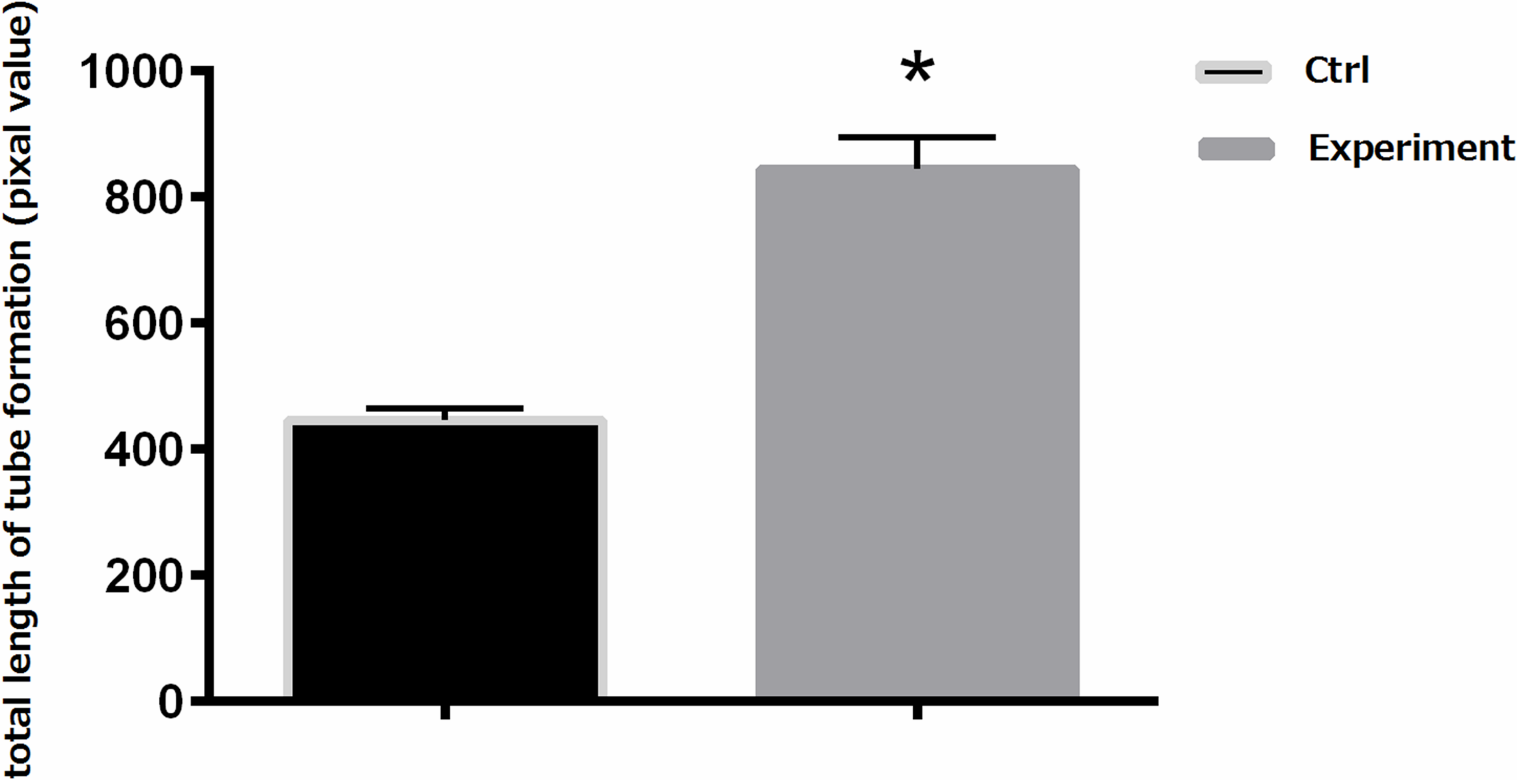
The total length of tube formation of HUVECs. The total length of tube formation in both the experimental groups and control groups were analyzed by Image-Pro Plus 6.0 software. The figure shows that the total length of tube formation in the experimental groups was higher than the control groups (P<0.05).

To further elucidate the bioactivity of VEGF released by nanographene coated screws, Matrigel tube formation was investigated in vitro. When cultured with cumulative 9-day extracts of VEGF-loaded nanographene coated screws for 12 hours, the average length of tubular structures formed by HUVECs was significantly increased (P<0.05), as compared with the control groups (**Figs 6 and 7**). The result demonstrated that the released VEGF still had bioactivity.

### X-ray examination

**Fig 8:**
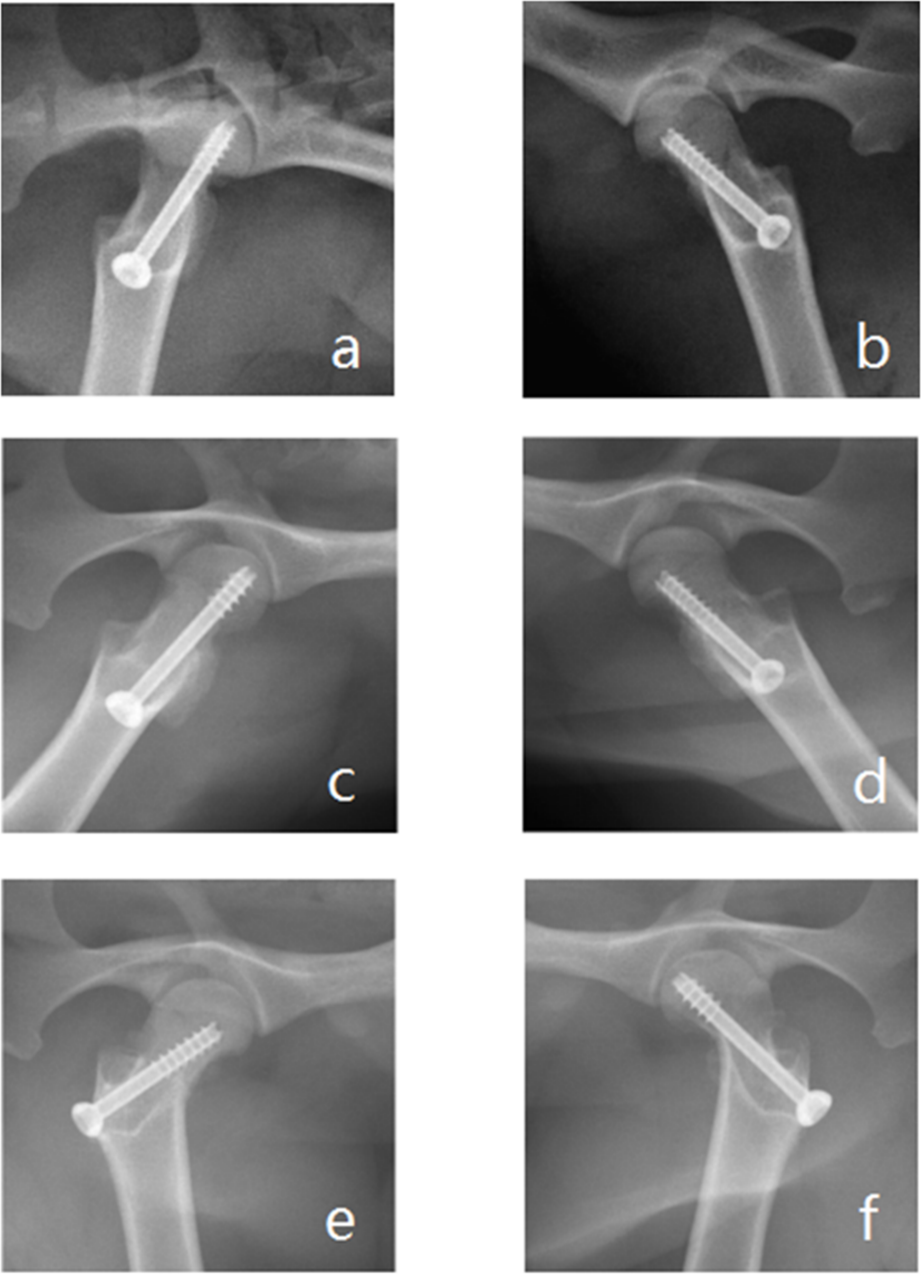
X-ray examination. After surgery (day 1), the fractures in both the experimental group (a) and control group (b) were clear and received good reduction. By 8 weeks postoperatively, the fracture line disappeared in the experimental groups (c), and BMD in the fracture site was similar with surrounding bone tissue. In the control group (d), the fracture line was blurred and BMD was lower than the bone tissue around the fracture site. At 12 weeks after surgery, the fracture lines in both groups disappeared. The BMD of the fracture site in the experimental group (e) was consistent with the surrounding bone tissue; however, the BMD in the control group (f) was still lower than the neighboring bone tissue.

After surgery, all objects had clear fracture lines and received good reduction (**Fig 8A and 8B**). By 8 weeks postoperatively, the fracture lines in the experimental groups disappeared and the bone density was similar to the bone tissues around the fracture site (**Fig 8C**) compared with the fracture line blurred in the control groups and a lower bone density in the fracture site (**Fig 8D**). By 12 weeks, the fracture was completely healed in the experimental groups with no apparent difference in the bone density of the fracture site as compared with the nearby normal bone tissue (**Fig 8E**). In the control groups, the fracture lines were almost invisible as well, and the bone density of the fracture site was still lower than the normal bone tissue around it (**Fig 8F**).

### 2D reconstructed images

**Fig 9:**
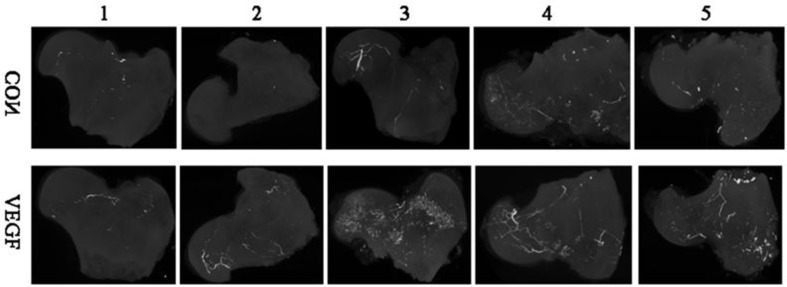
The MIP of microangiography of the femoral heads. The MIP of microangiography of the femoral heads shows that the number and branches of microvessels in the experimental group (VEGF) was higher than the control group (CON).

After 12 weeks of surgery, the MIP images showed that there were several microvessels in the femoral heads of the experimental groups (**Fig 9A**) connected with the vessels of great trochanter of the femur through the femoral neck, and they distributed more evenly and broadly compared with the control groups. In the control groups (**Fig 9B**), the numbers and branches of microvessels in the femoral heads were lower than the experimental groups, and there was no obvious microvessels of the femoral heads connected with the surrounding vessels.

### 3D reconstructed images

**Fig 10:**
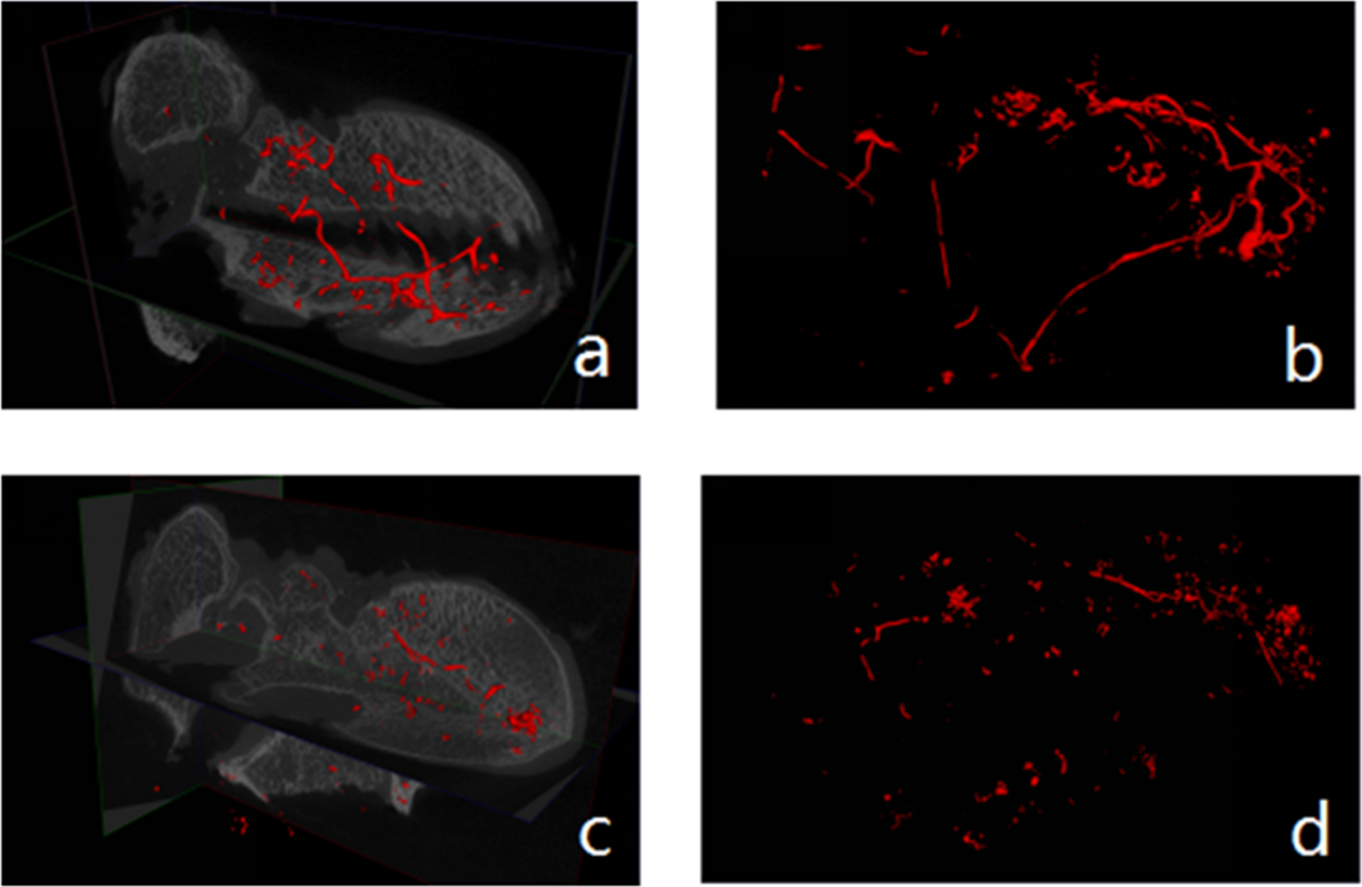
3D images of microvessels and bone structure. The three-dimensional images of microvessels and bone structure (left) in the femoral heads could be displayed simultaneously through Isosurface of Micro View software. By choosing the appropriate CT values, the information of microvessels (right) could be extracted separately. The images demonstrated that the microvessels in the experimental group (a,b) were more evenly and broadly distributed than in the control group (c,d).

The 3D images of the microvessels within the femoral heads were reconstructed and customized to red by Isosurface of Micro View. The spatial relationship between the bone microstructure and microvessels could be observed from the coronal and sagittal planes (**Fig 10A and 10C**). By choosing the appropriate threshold (3000 HU), microvessels could be extracted and displayed directly without the surrounding bone structure (**Fig 10B and 10D**). The results of the 3D-reconstructed images of the microvessels further demonstrated that the numbers and branches of microvessels in the femoral heads of the experimental groups (**Fig 10A and 10B**) were more than the control groups (**Fig 10C and 10D**).

### Advanced bone analysis of femoral heads

Bone structural parameters are shown in **Table 2**. The BMD of the femoral heads of the experimental groups was significantly higher than the control groups (408.6 ± 39.9 vs. 310.6 ± 28.7, P<0.05). The Tb.Th of the two groups had no statistically significant difference despite the fact that there was an increasing trend in the experimental groups.

**Table 2:** The BMD and Tb.Th of the femoral heads after micro-CT analysis at 12 weeks postoperatively (n = 5).

Group	Control	Experiment
**BMD(mg/cc)**	310.6±28.70	408.6±39.94
**Tb.Th(μm)**	126.6±9.36	164.4±19.54

The BMD of the femoral heads of the experimental groups was significantly higher than the control groups. The Tb.Th of the two groups had no statistically significant difference despite the fact that there was an increasing trend in the experimental groups.

### Histopathological evaluation

**Fig 11:**
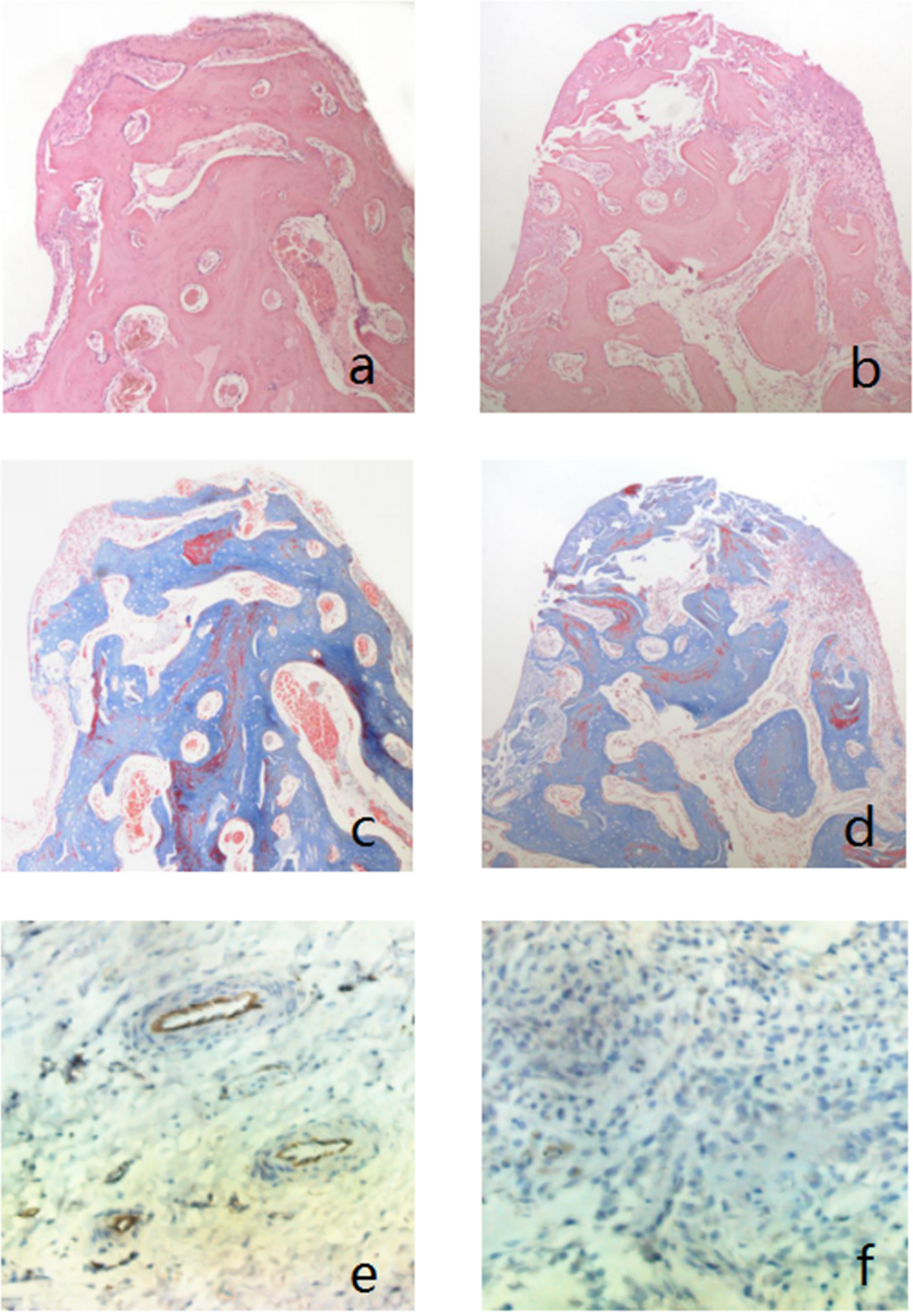
Histopathological evaluation of the femoral heads. HE staining (x100), Masson’s trichrome staining (x100), and CD31 immunohistochemical staining (x200) of the femoral heads at 12 weeks after surgery. a, c, e experimental groups; b, d, f control groups. a, b, c, d show the bone tissue adjacent to the screw trajectory.

Compared with the control groups, the width and area of the bone trabecula of the experimental groups showed in HE staining were higher at 12 weeks postoperatively (**Fig 11A and 11B**). However, the results had no statistically significant difference (P>0.05) (**Table 3**). The images of Masson’s trichrome stain (**Fig 11C and 11D**) showed that there was more red-stained mature bone observed in the femoral heads of the experimental groups than the control groups. New bone formation area (%) in the experimental femoral heads (**Table 4**) was more than the control groups (p<0.05). CD31 immunohistochemical staining (**Fig 11E and 11F**) demonstrated that the number of CD31 positive cells and microvessels of experimental groups (**Table 5**) was significantly higher than the control groups (P<0.05).

**Table 3:** Data analysis of trabecular bone of HE staining (n = 5). ($\bar{x}$±s).

Group	Control	Experiment
**Percent trabecular area (%)**	43.68±3.89	50.80±3.28
**Trabecular width (μm)**	129.00±10.17	146.80±19.45

Compared with the control groups, the width and area of the bone trabecula of the experimental groups showed in HE staining were higher at 12 weeks postoperatively. However, the results had no statistically significant difference.

**Table 4:** Data analysis of new bone formation area (%) of Masson’s trichrome staining (n = 5). ($\bar{x}$±s).

Group	Control	Experiment
**New bone formation area (%)**	8.31±1.60	17.30±1.50

New bone formation area (%) in the experimental femoral heads was larger than the control groups.

**Table 5:** Microvessel density of CD31 immunohistochemical staining (n = 5). ($\bar{x}$±s).

Group	Control	Experiment
**Microvessel density (MVD)**	61.20±9.39	113.40±12.50

CD31 immunohistochemical staining demonstrated that the number of CD31 positive cells and microvessels of experimental groups was significantly higher than the control groups.

## Discussion

Previous studies have proven that the severity of fracture displacement and internal fixation avascular necrosis incidence were closely related: the higher the degree of fracture displacement, the more complete damage to the blood supply of the femoral head, and the higher the incidence of postoperative ANFH [17,18]. A number of recent studies have shown that the choice of surgical treatment for femoral neck fracture should be based on the remaining blood supply of the femoral head after injury rather than relying solely on the severity of the fracture displacement [19,20]. This suggests that early recovery of the femoral head blood supply could be the key to preventing ANFH after internal fixation.

We chose VEGF as the delivery drug for the treatment of femoral neck fracture. VEGF, as the most important regulatory factor of angiogenesis, is involved in all processes of angiogenesis [6,7,21]. VEGF also played a crucial role in the progress of bone growth as well as fracture repair [10,11,22]. Our experiment results demonstrated that the microvessel density and BMD of the femoral heads in the experimental sides increased compared with the control sides, indicating that VEGF-loaded nanographene coated screws could accelerate fracture healing and promote blood vessel repair. To load VEGF into the fracture site effectively, we prepared nanographene coating as the drug delivery system. Nanographene, as a novel biomaterial, has been proved to have good biocompatibility [23]. Also, Kim et al. [24] and Li et al. [25] reported that nanographene could be conductive of the adhesion and differentiation of adipose-derived MSCs and neural stem cells. Nanographene material can be applied to the surface of any shape of materials as a single atom sheet to enhance the materials’ performance, such as biocompatibility and cell adhesion [26]. As reported nanographene has shown the advantage of little cytotoxicity while with the ability sustained release for many days[27,28].Our experimental results showed that the nanographene could be successfully applied as a coating on the surface of the internal fixing screws and that it did not affect the biocompatibility and surface characteristics of the screws. The nanographene coating could absorb a certain amount of VEGF, demonstrating effective sustained release of VEGF. As a simple and effective drug delivery system, nanographene coating could deliver VEGF to the fracture site directly in the treatment of femoral neck fracture.

Microangiography is one of the most intuitive methods in the assessment of the blood supply recovery of the femoral head after injury. We carried out microangiography by abdominal aorta and inferior vena cava catheter, which is a clear, simple approach that is easy to repeat. The diameter of subnanometer barium sulfate particles is smaller than 500 nm and can be easily injected into microvessels of bone. The CT value of the subnanometer barium sulfate, as the vascular contrast agent, is greater than 3000 HU. However, the CT value of cancellous bone CT is generally less than 1500 HU. This CT value difference could be distinguished between microvessels and bone microstructure in femoral heads. Thus, samples could be scanned by micro-CT without decalcification, so as to keep all the vascular and bony information in the femoral heads. After image reconstruction, microvessels and bone microstructure within the femoral head could be displayed simultaneously and analyzed from any two-dimensional and three-dimensional level through Micro View software. In addition, compared with Microfil [29] and Angiofil [30], subnanometer barium sulfate is a highly economical and suitable agent for the angiography of medium and large animals. Our experiment results showed that subnanometer barium sulfate could be successfully used as a contrast agent for the microangiography of beagles. After micro-CT scanning, the distribution of microvessels could be displayed clearly from two- and three-dimensional aspects.

The main limitations in our study were that we reduced the fixation time to 12 weeks. If it could be extended to 6 months, there might be a better result. In our study, we testified that nanographene coating had good biocompatibility, but we did not study the rest of material characterisation of it, such as adhesivity.Otherwise,this animal model could not reflect the real condition that is seen in human fracture healing process and may account for the excellent healing of experimental animals in this study.

## Conclusion

In conclusion, our experiment proved that VEGF-loaded nanographene coated internal fixation screws could promote new bone formation and the recovery of blood supply in the fracture healing process. As a new contrast agent, combined with micro-CT scanning, subnanometer barium sulfate, as a new contrast agent, could clearly show microvessels in femoral heads and make the quantitative analysis of microvessels and bone microstructure possible.

## Supporting information

S1 Dataset (XLS)

## References

[1] Papakostidis C, Panagiotopoulos A, Piccioli A, Giannoudis PV. Timing of internal fixation of femoral neck fractures. A systematic review and meta-analysis of the final outcome. INJURY. 2015; 46(3): 459–466. doi: 10.1016/j.injury.2014.12.025 25616675 10.1016/j.injury.2014.12.025

[2] Zhao D, Wang Z, Wang B, Qiu X, Liu B, Yang L, et al Revascularization of the femoral head by anastomosis of superior retinacular vessels for the treatment of femoral neck fracture: A case report. MICROSURG. 2016 2 20 doi: 10.1002/micr.30029 26895831 10.1002/micr.30029

[3] Davidovitch R I, Jordan C J, Egol K A, Vrahas MS. Challenges in the treatment of femoral neck fractures in the nonelderly adult. J Trauma.2010; 68(1): 236–242. doi: 10.1097/TA.0b013e3181c428ce 20065780 10.1097/TA.0b013e3181c428ce

[4] Gothard D, Smith E L, Kanczler J M, Rashidi H, Qutachi O, Henstock J, et al Tissue engineered bone using select growth factors: A comprehensive review of animal studies and clinical translation studies in man. Eur Cell Mater. 2014; 28: 166–207, 207–208. 25284140 10.22203/ecm.v028a13

[5] Keramaris N C, Calori G M, Nikolaou V S, Schemitsch EH, Giannoudis PV. Fracture vascularity and bone healing: a systematic review of the role of VEGF. INJURY.2008; 39 Suppl 2: S45–S57. doi: 10.1016/S0020-1383(08)70015-9 18804573 10.1016/S0020-1383(08)70015-9

[6] Isner J M, Pieczek A, Schainfeld R, Blair R, Haley L, Asahara T, et al Clinical evidence of angiogenesis after arterial gene transfer of phVEGF165 in patient with ischaemic limb. The Lancet. 1996; 348(9024): 370–374. doi: 10.1016/S0140-6736(96)03361-2 10.1016/s0140-6736(96)03361-2 8709735

[7] Holash J, Maisonpierre P C, Compton D, Boland P, Alexander CR, Zagzag D, et al Vessel cooption, regression, and growth in tumors mediated by angiopoietins and VEGF. SCIENCE. 1999; 284(5422): 1994–1998. 10373119 10.1126/science.284.5422.1994

[8] Stegen S, van Gastel N, Carmeliet G. Bringing new life to damaged bone: the importance of angiogenesis in bone repair and regeneration. BONE. 2015; 70: 19–27. doi: 10.1016/j.bone.2014.09.017 25263520 10.1016/j.bone.2014.09.017

[9] Clarkin C E, Gerstenfeld L C. VEGF and bone cell signalling: an essential vessel for communication? CELL BIOCHEM FUNCT. 2013; 31(1): 1–11. doi: 10.1002/cbf.2911 23129289 10.1002/cbf.2911

[10] Hu K, Olsen B R. Osteoblast-derived VEGF regulates osteoblast differentiation and bone formation during bone repair. J CLIN INVEST. 2016; 126(2): 509–526. doi: 10.1172/JCI82585 26731472 10.1172/JCI82585 PMC4731163

[11] Street J, Bao M, DeGuzman L, Bunting S, Peale FV Jr, Ferrara N, et al Vascular endothelial growth factor stimulates bone repair by promoting angiogenesis and bone turnover. Proc Natl Acad Sci U S A. 2002; 99(15): 9656–9661. doi: 10.1073/pnas.152324099 12118119 10.1073/pnas.152324099 PMC124965

[12] Lee C, Wei X, Kysar J W, Hone J. Measurement of the elastic properties and intrinsic strength of monolayer graphene. SCIENCE. 2008; 321(5887): 385–388. doi: 10.1126/science.1157996 18635798 10.1126/science.1157996

[13] Chae H K, Siberio-Perez D Y, Kim J, Go Y, Eddaoudi M, Matzger AJ, et al A route to high surface area, porosity and inclusion of large molecules in crystals. NATURE. 2004; 427(6974): 523–527. doi: 10.1038/nature02311 14765190 10.1038/nature02311

[14] Ramanathan T, Abdala A A, Stankovich S, Dikin DA, Herrera-Alonso M, Piner RD, et al Functionalized graphene sheets for polymer nanocomposites. NAT NANOTECHNOL. 2008; 3(6): 327–331. doi: 10.1038/nnano.2008.96 18654541 10.1038/nnano.2008.96

[15] La WG, Park S, Yoon H H, Jeong GJ, Lee TJ, Bhang SH, et al Delivery of a therapeutic protein for bone regeneration from a substrate coated with graphene oxide. SMALL. 2013; 9(23): 4051–4060. doi: 10.1002/smll.201300571 23839958 10.1002/smll.201300571

[16] Takahashi Y, Cleary K R, Mai M, Kitadai Y, Bucana CD, Ellis LM. Significance of vessel count and vascular endothelial growth factor and its receptor (KDR) in intestinal-type gastric cancer. CLIN CANCER RES. 1996; 2(10): 1679–1684. 9816116

[17] Ehlinger M, Moser T, Adam P, Bierry G, Gangi A, de Mathelin M, et al Early prediction of femoral head avascular necrosis following neck fracture. Orthop Traumatol Surg Res. 2011; 97(1): 79–88. doi: 10.1016/j.otsr.2010.06.014 21087905 10.1016/j.otsr.2010.06.014

[18] Bachiller F G, Caballer A P, Portal L F. Avascular necrosis of the femoral head after femoral neck fracture. Clin Orthop Relat Res. (399): 87–109. 12011698 10.1097/00003086-200206000-00012

[19] Yuan H F, Shen F, Zhang J, Shi HC, Gu YS, Yan ZQ. Predictive value of single photon emission computerized tomography and computerized tomography in osteonecrosis after femoral neck fracture: a prospective study. INT ORTHOP. 2015; 39(7): 1417–1422. doi: 10.1007/s00264-015-2709-7 25711398 10.1007/s00264-015-2709-7

[20] Kumar M N, Belehalli P, Ramachandra P. PET/CT study of temporal variations in blood flow to the femoral head following low-energy fracture of the femoral neck. ORTHOPEDICS. 2014; 37(6): e563–e570. doi: 10.3928/01477447-20140528-57 24972438 10.3928/01477447-20140528-57

[21] Ferrara N. VEGF-A: a critical regulator of blood vessel growth. EUR CYTOKINE NETW. 2009; 20(4): 158–163. doi: 10.1684/ecn.2009.0170 20167554 10.1684/ecn.2009.0170

[22] Mayr-Wohlfart U, Waltenberger J, Hausser H, Kessler S, Gunther KP, Dehio C, et al Vascular endothelial growth factor stimulates chemotactic migration of primary human osteoblasts. BONE. 2002; 30(3): 472–477. 11882460 10.1016/s8756-3282(01)00690-1

[23] Sun X, Liu Z, Welsher K, Robinson JT, Goodwin A, Zaric S, et al Nano-Graphene Oxide for Cellular Imaging and Drug Delivery. NANO RES. 2008; 1(3): 203–212. doi: 10.1007/s12274-008-8021-8 20216934 10.1007/s12274-008-8021-8 PMC2834318

[24] Kim J, Choi K S, Kim Y, Lim KT, Seonwoo H, Park Y, et al Bioactive effects of graphene oxide cell culture substratum on structure and function of human adipose-derived stem cells. J BIOMED MATER RES A. 2013; 101(12): 3520–3530. doi: 10.1002/jbm.a.34659 23613168 10.1002/jbm.a.34659

[25] Li N, Zhang Q, Gao S, Song Q, Huang R, Wang L, et al Three-dimensional graphene foam as a biocompatible and conductive scaffold for neural stem cells. Sci Rep. 2013; 3: 1604 doi: 10.1038/srep01604 23549373 10.1038/srep01604 PMC3615386

[26] Lee W C, Lim C H, Shi H, Tang LA, Wang Y, Lim CT, et al Origin of enhanced stem cell growth and differentiation on graphene and graphene oxide. ACS NANO. 2011; 5(9): 7334–7341. doi: 10.1021/nn202190c 21793541 10.1021/nn202190c

[27] Zhang X, Yang C, Zhou J, Huo M.Somatostatin Receptor-Mediated Tumor-Targeting Nanocarriers Based on Octreotide-PEG Conjugated Nanographene Oxide for Combined Chemo and Photothermal Therapy.Small. 2016;12(26):3578–90. doi: 10.1002/smll.201600618 27244649 10.1002/smll.201600618

[28] Li T, Wu L, Zhang J, Xi G, Pang Y, Wang X, Chen T.Hydrothermal Reduction of Polyethylenimine and Polyethylene Glycol Dual-Functionalized Nanographene Oxide for High-Efficiency Gene Delivery.ACS Appl Mater Interfaces. 2016;8(45):31311–31320. doi: 10.1021/acsami.6b09915 27813400 10.1021/acsami.6b09915

[29] Nyangoga H, Mercier P, Libouban H, Basle MF, Chappard D.Three-dimensional characterization of the vascular bed in bone metastasis of the rat by microcomputed tomography (MicroCT). PLOS ONE. 2011; 6(3): e17336 doi: 10.1371/journal.pone.0017336 21464932 10.1371/journal.pone.0017336 PMC3065464

[30] Grabherr S, Hess A, Karolczak M, Thali MJ, Friess SD, Kalender WA, et al Angiofil-mediated visualization of the vascular system by microcomputed tomography: a feasibility study. Microsc Res Tech. 2008; 71(7): 551–556. doi: 10.1002/jemt.20585 18393302 10.1002/jemt.20585

