# Correction: The treatment of femoral neck fracture using VEGF-loaded nanographene coated internal fixation screws

**DOI:** 10.1371/journal.pone.0191143

**Published:** 2018-01-09

**Authors:** Shuo Li, Hengfeng Yuan, Jianfeng Pan, Wenshuai Fan, Liang Zhu, Zuoqin Yan, Changan Guo

There is an error in the affiliation for authors Shuo Li, Hengfeng Yuan, Jianfeng Pan, Wenshuai Fan, Liang Zhu, Zuoqin Yan and Changan Guo. The affiliation should be:

Department of Orthopedics, Zhongshan Hospital, Fudan University, Shanghai, China.
